# Genetic Variants in Oxytocinergic System Genes and Their Association with Postpartum Depression Susceptibility

**DOI:** 10.3390/ijms26052129

**Published:** 2025-02-27

**Authors:** Livia Ciolac, Nicoleta Ioana Andreescu, Simona Sorina Farcaș, Elena Silvia Bernad, Anca Tudor, Dumitru-Răzvan Nițu, Daian-Ionel Popa, Anca-Laura Maghiari, Marius Lucian Craina

**Affiliations:** 1 Doctoral School, Faculty of General Medicine, “Victor Babes” University of Medicine and Pharmacy Timisoara, 300041 Timisoara, Romania; livia.ciolac@umft.ro (L.C.); daian-ionel.popa@umft.ro (D.-I.P.); 2 Department of Microscopic Morphology, Discipline of Genetics, Genomic Medicine Centre, “Victor Babes” University of Medicine and Pharmacy Timisoara, 2 Eftimie Murgu Square, 300041 Timisoara, Romania; andreescu.nicoleta@umft.ro (N.I.A.); farcas.simona@umft.ro (S.S.F.); 3 Department of Obstetrics and Gynecology, Faculty of Medicine, “Victor Babes” University of Medicine and Pharmacy Timisoara, 300041 Timisoara, Romania; bernad.elena@umft.ro (E.S.B.); nitu.dumitru@umft.ro (D.-R.N.); mariuscraina@umft.ro (M.L.C.); 4 Ist Clinic of Obstetrics and Gynecology, “Pius Brinzeu” County Clinical Emergency Hospital, 300723 Timisoara, Romania; 5 Center for Laparoscopy, Laparoscopic Surgery and In Vitro Fertilization, “Victor Babes” University of Medicine and Pharmacy Timisoara, 300041 Timisoara, Romania; 6 Department of Biostatistics and Medical Informatics, “Victor Babes” University of Medicine and Pharmacy Timisoara, 300041 Timisoara, Romania; 7 Research Center for Medical Communication, “Victor Babes” University of Medicine and Pharmacy Timisoara, Eftimie Murgu Square No. 2, 300041 Timisoara, Romania; 8 Department I—Discipline of Anatomy and Embryology, Faculty of Medicine, “Victor Babes” University of Medicine and Pharmacy Timisoara, 2nd Eftimie Murgu Square, 300041 Timisoara, Romania; boscu.anca@umft.ro

**Keywords:** oxytocin, oxytocin receptor, EPDS, postpartum depression, single nucleotide polymorphism, genetic variation

## Abstract

One of the most frequent forms of maternal morbidity following childbirth is postpartum depression. Postpartum depression (PPD), a disabling condition as a major public health concern, has a significant negative impact on the child’s emotional, mental as well as intellectual development if left undiagnosed and untreated, which can later have long-term complications. The oxytocin system is an excellent candidate gene system in the maternal context. Differences in vulnerability of mothers for the onset of postpartum psychiatric disorders could be influenced by individual differences in the genetic profile of each one. In this original research, we aimed to explore if there are any possible contributions of genetic variation on both the oxytocin receptor gene (OXTR) and the oxytocin gene (OXT) to the occurrence of postpartum depression, aiming to provide the latest evidence and determine which genetic polymorphisms significantly create a susceptibility for this condition. A total of 100 mothers were preliminarily genotyped before they completed the Edinburgh Postnatal Depression Scale Questionnaire (EPDS) at 6 weeks postpartum. DNA was extracted from peripheral blood samples of the participants (N = 100) and evaluated for the oxytocin gene (OXT_rs2740210; OXT_rs4813627) and oxytocin receptor gene (OXTR_ rs237885) single nucleotide polymorphisms. The results highlighted a significant interaction between the oxytocin OXT_rs2740210 genotype and maternal postpartum depression in mothers with the CC genotype but not in those with AA/AC genotypes. This reveals that an interaction of vulnerable genotypes (CC genotype of OXT_rs2740210, C allele in genotype of OXT_rs2740210, G allele in genotype of OXT_rs4813627) with an environmental burden or other risk factors would predispose the mothers to develop postpartum depression. We found no significant association between the interaction effect of the oxytocin receptor gene OXTR_rs237885 genotype depending on the occurrence of maternal postpartum depression. These findings prove the implication of the oxytocinergic system gene variants in vulnerability for postpartum depression and indicate the need for future studies adopting a multilevel approach in order to increase understanding.

## 1. Introduction

Childbirth represents for women a time of great vulnerability to becoming mentally unwell, with postpartum mood disorders representing the most frequent form of maternal morbidity following delivery [1]. These affective disorders range in severity from the early maternity blues to postpartum psychosis, a serious state affecting less than 1% of mothers and usually requiring hospitalization [1,2]. Along this spectrum is postpartum depression (PPD), a disabling condition as a major public health concern suffered by approximately 13% of women in the United States and 17% of women globally [3]. Not only is maternal depression among the most prevalent psychiatric disorders [4] but the majority of cases also go undiagnosed, and even mild or moderate symptoms of depression are deleterious for mothers and potentially harmful to the infant and the family [3]. The inception rate is greatest in the first 12 weeks and, while residual depressive symptoms are common, up to 50% of mothers will remain clinically depressed at 6 months postpartum [1]. Given the consequences of postpartum depression and its symptoms, hundreds of studies have examined biological and psychological risk factors. Behavior genetics studies have revealed the role of genetics in depression, with heritability estimates ranging between 40 and 70%, although no single genes have been identified yet [5]. Additionally, studies documenting gene–environment interactions have begun to demonstrate that individuals tend to inherit genetic predispositions for a particular disorder, rather than inheriting any particular disorder itself [5].

The oxytocin neurobiological system could lie behind the development of maternal postpartum depression and its cross-generational effects. The oxytocin system is an excellent candidate gene system in the maternal context [6]. The neurohormone oxytocin (OXT), mediated through its specific receptor (OXTR), is involved in the regulation of social behavior and social cognition [7]. Oxytocin, a nine-amino-acid neuropeptide synthesized in the hypothalamus, has long been implicated in birth and lactation, and recent research highlighted its role in providing the neurohormonal substrate for mammalian social bonding [8]. Animal studies have shown that the neurochemical organization of infant brain oxytocin is shaped in early life through patterns of maternal behavior, such as licking and grooming [9]. This extra-genomic cross-generational transmission defines a biobehavioral feedback loop: maternal oxytocin determines the mother’s caregiving behavior, which in turn shapes the infant’s oxytocin through species-typical parenting behavior [10]. Human studies similarly demonstrate the involvement of oxytocin in parenting behavior [11], and show that parental oxytocin influences the infant’s oxytocin system [12]. Numerous studies have shown disruptions to the oxytocin system in depression, and thus, addressing its role in maternal depression may be a fruitful area of research and potential intervention [4]. The oxytocin system, as quantified by salivary and plasma levels of the peptide and single nucleotide polymorphisms (SNPs) in both the genes for the ligand and for the receptor, has been associated with social and emotional behaviors [13]. Several reports have focused on the effects of oxytocin ligand gene polymorphisms on maternal behavior in the early life of the infant, finding associations between oxytocin single nucleotide polymorphisms and infant-directed speech and instrumental care [6,14]. Oxytocin single nucleotide polymorphisms also associate with breastfeeding duration [6,14]. Similarly, single nucleotide polymorphisms in the oxytocin receptor gene are also implicated in maternal behavior, associating with affectionate maternal touch [4], maternal depression [4,15], and quality of early maternal care [16]. Plasma oxytocin during the third trimester of pregnancy predicts maternal postpartum depressive symptoms [17], and lower plasma oxytocin in the first trimester predicts postpartum depressive symptoms and a low level of attachment behavior [8].

Given the limitations of current theories of depression and anxiety, which emphasize the roles of serotonin and norepinephrine, research on oxytocin may provide a new direction for understanding these conditions [4].

Differences in vulnerability of mothers for the onset of postpartum psychiatric disorders could be influenced by individual differences in the genetic profile of each one. The role of genetic variation tends to be largely unexplored in the study of postpartum mood and, furthermore, of psychiatric disorders with onset in the postpartum period. In this original research, we aimed to explore if there are any possible contributions of genetic variation on both the oxytocin receptor gene (OXTR) and the oxytocin gene (OXT) to the occurrence of postpartum depression, aiming to provide the latest evidence and determine which genetic polymorphisms significantly creates a susceptibility for this condition. To the best of our knowledge, there has not been any assessment of the strength of OXT rs4813627, OXTR rs237885, or OXTR rs2740210 polymorphism associated with the development of postpartum depression; therefore, our research is the first report in the literature.

## 2. Results

A total of 100 mothers were investigated individually and completed the Edinburgh Postnatal Depression Scale Questionnaire (EPDS) at 6 weeks postpartum. A cutoff score ≥13 was used to detect a major postpartum depressive disorder, thus, 16% (16 patients) had depressive disorder, while 84% (84 patients) had no depressive disorder.

Nominal variables were represented by absolute and relative frequency, n (%), while numerical variables (quantitative variables) were represented by mean, standard deviation and median (interquartile range).

### 2.1. OXT_rs2740210 Genotyping

In the evaluation process for single nucleotide polymorphisms of oxytocin gene OXT_rs2740210, no results were obtained for two samples from the 100 included; therefore, they were excluded from the analysis (N = 98). Genotyping of OXT_rs2740210 revealed that 44 mothers had the CC genotype (44.9%), 41 mothers had the AC genotype (41.8%), and 13 mothers had the AA genotype (13.3%).

There was observed a significant association between the occurrence of maternal postpartum depression and the oxytocin OXT_rs2740210 genotype (Chi2 test, *p* < 0.001) (Table 1).

**Figure 1:**
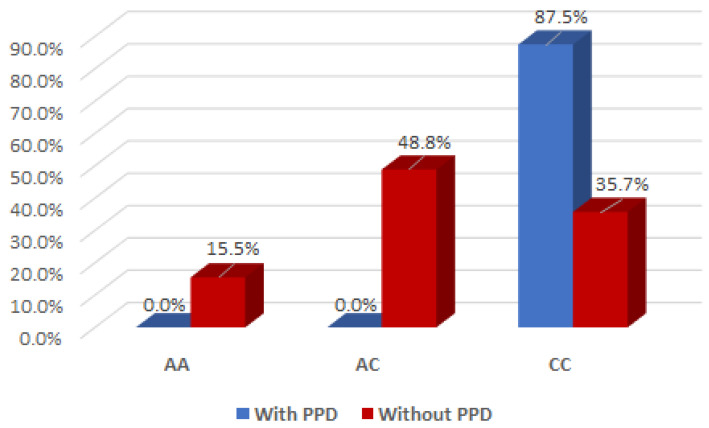
Interaction effect of the oxytocin gene OXT_rs2740210 genotype and the occurrence of maternal postpartum depression.

The association between postpartum depression and the oxytocin OXT_rs2740210 genotype was evident in mothers with the CC genotype (Yate’s corrected Chi2 test, *p* < 0.001), but not in those with AA/AC genotypes (Figure 1).

It is of interest to note that there was a significant interaction effect between mothers with the AC genotype for OXT_rs2740210 and the absence of clinical postpartum depression (Yate’s corrected Chi2 test, *p* < 0.001), indicating that the presence of the AC genotype for OXT_rs2740210 could be considered a protective factor for the occurrence of postpartum depression (Figure 1).

Then, we examined any other interaction effect between the OXT_rs2740210 genotype, depending on the occurrence of maternal postpartum depression, and other variables of study participants by applying multiple analysis (Table 1). We found no other significant interactions.

**Figure 2:**
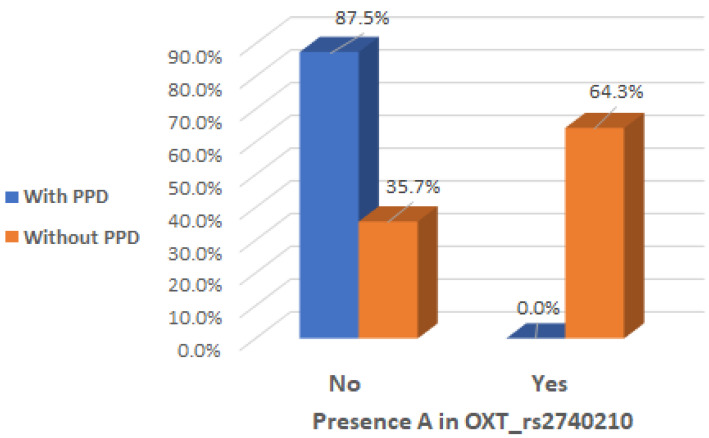
Presence of A allele in the OXT_rs2740210 genotype depending on the occurrence of postpartum depression.

It is of interest to observe that there was a significant interaction effect between mothers with presence of A allele in the genotype of OXT_rs2740210 and the absence of clinical postpartum depression (Chi2 test, *p* < 0.001), indicating that the presence of A allele in the genotype of OXT_rs2740210 could be considered a protective factor (Table 2 and Figure 2).

**Figure 3:**
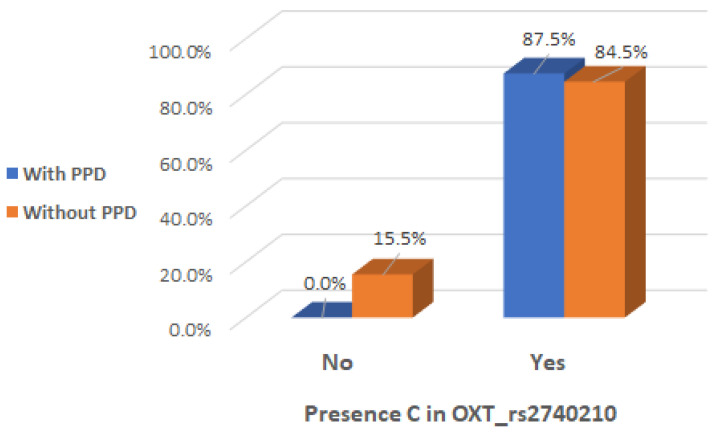
Presence of C allele in the OXT_rs2740210 genotype depending on the occurrence of postpartum depression.

There was a significant interaction effect between mothers with presence of C allele in the genotype of OXT_rs2740210 and the occurrence of maternal postpartum depression (Chi2 test, *p* = 0.002), indicating that the presence of C allele in the genotype of OXT_rs2740210 could be considered a risk factor (Table 2 and Figure 3).

**Figure 4:**
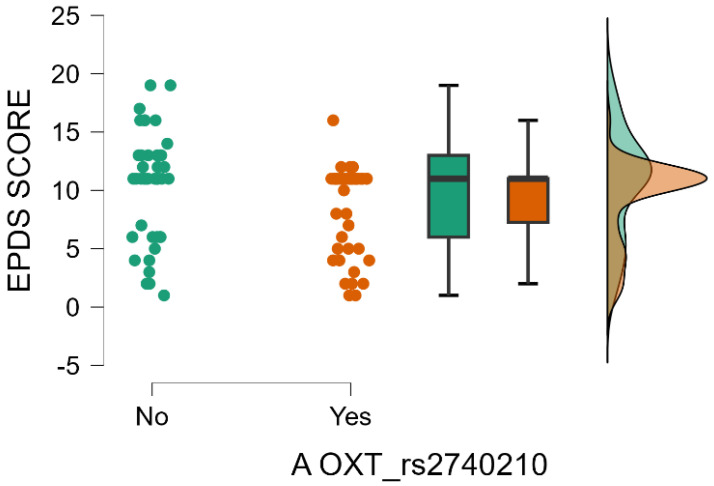
Presence of A allele in the OXT_rs2740210 genotype depending on EPDS score.

Furthermore, it is noteworthy that there is a significant interaction effect between the presence of A allele in the OXT_rs2740210 genotype and the mean value of EPDS score. The mean value of EPDS score is significantly low for the mothers with presence of A allele in the OXT_rs2740210 genotype (Mann–Whitney test, *p* = 0.017) (Table 3 and Figure 4).

There does not exist any significant interaction effect between the presence of C allele in the OXT_rs2740210 genotype and the mean value of the EPDS score. The mean value of the EPDS score is not significantly increased for the mothers with presence of C allele in the OXT_rs2740210 genotype (Mann–Whitney test, *p* = 0.161) (Table 3).

### 2.2. OXT_rs4813627 Genotyping

In the evaluation process for single nucleotide polymorphisms of the oxytocin gene OXT_rs4813627, no results were obtained for 1 sample from the 100 included. Therefore, it was excluded from the analysis (N = 99).

Genotyping of OXT_rs4813627 revealed that 52 mothers had the AG genotype (52.53%), 24 mothers had the AA genotype (24.24%), and 23 mothers had the GG genotype (23.23%).

There was not observed any significant association between the occurrence of maternal postpartum depression and the oxytocin OXT_rs4813627 genotype (Table 4).

We examined the interaction effect of the OXT_rs4813627 genotype, depending on maternal history of postpartum depressive disorder and found a significant association (Chi2 test, *p* = 0.041) (Table 4).

**Figure 5:**
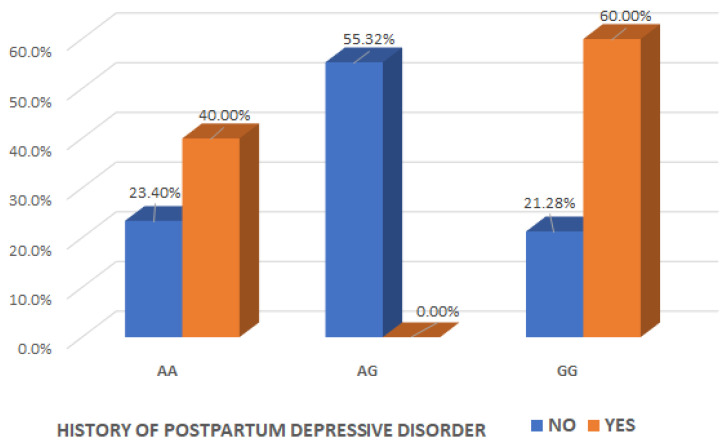
Interaction effect of the oxytocin gene OXT_rs4813627 genotype and the maternal history of postpartum depressive disorder.

It is of interest to observe that there was a significant interaction effect between mothers with the AG genotype for OXT_rs4813627 and the absence history of maternal postpartum depressive disorder (Chi2 test, *p* = 0.040), indicating that the presence of the AG genotype for OXT_rs4813627 could be considered a protective factor for the occurrence of postpartum depression (Table 4 and Figure 5).

**Figure 6:**
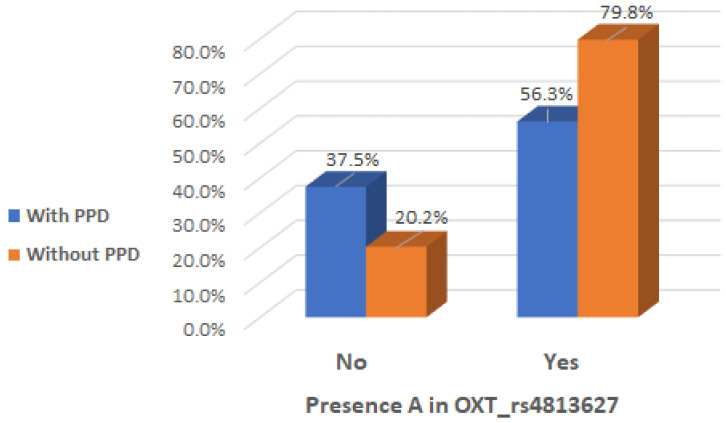
Presence of A allele in OXT_rs4813627 genotype depending on the occurrence of postpartum depression.

It is noteworthy that there was a significant interaction effect between mothers with the presence of A allele in the genotype of OXT_rs4813627 and the absence of clinical postpartum depression (Chi2 test, *p* = 0.019), indicating that the presence of A allele in the genotype of OXT_rs4813627 could be considered a protective factor (Table 5 and Figure 6).

**Figure 7:**
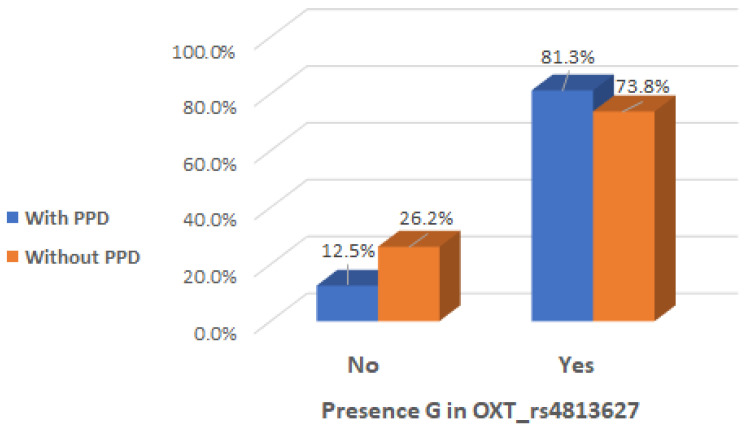
Presence of G allele in OXT_rs4813627 genotype depending on the occurrence of postpartum depression.

There was a significant interaction effect between mothers with presence of G allele in the genotype of OXT_rs4813627 and the occurrence of maternal postpartum depression (Chi2 test, *p* = 0.041), indicating that the presence of G allele in the genotype of OXT_rs4813627 could be considered a risk factor (Table 5 and Figure 7).

There does not exist any significant interaction effect between the presence of A allele in OXT_rs4813627 genotype and the mean value of EPDS score. The mean value of EPDS score is not significantly low for the mothers with presence of A allele in the OXT_rs4813627 genotype (Mann–Whitney test, *p* = 0.439) (Table 6).

There does not exist any significant interaction effect between the presence of G allele in the OXT_rs4813627 genotype and the mean value of EPDS score. The mean value of EPDS score is not significantly increased for the mothers with the presence of G allele in the OXT_rs4813627 genotype (Mann–Whitney test, *p* = 0.337) (Table 6).

### 2.3. OXTR_rs237885 Genotyping

In the evaluation process for single nucleotide polymorphisms of oxytocin receptor gene OXTR_rs237885, no results were obtained for 3 samples from the 100 included. Therefore, they were excluded from the analysis (N = 97).

Genotyping of OXTR_rs237885 revealed that 45 mothers had GT genotype (46.4%), 37 mothers had the GG genotype (38.1%) and 15 mothers had the TT genotype (15.5%).

We found no significant association between the interaction effect of oxytocin receptor gene OXTR_rs237885 genotype depending on the occurrence of maternal postpartum depression or any other variables of study participants, even after applying multiple analysis (Chi2 test; Kruskal-Wallis test; *p* > 0.05) (Table 7).

We found no significant increased interaction effect between mothers with presence of G allele in the genotype of OXTR_rs237885 and the absence of clinical postpartum depression (Chi2 test, *p* = 0.687) (Table 8).

There is no significant increased interaction effect between mothers with the presence of T allele in the genotype of OXTR_rs237885 and the onset of postpartum depression (Chi2 test, *p* = 0.650) (Table 8).

There does not exist any significant interaction effect between the presence of G allele in the OXTR_rs237885 genotype and the mean value of the EPDS score. The mean value of the EPDS score is not significantly low for the mothers with presence of G allele in OXTR_rs237885 genotype (Mann–Whitney test, *p* = 0.439) (Table 9).

There does not exist any significant interaction effect between the presence of T allele in the OXTR_rs237885 genotype and the mean value of the EPDS score. The mean value of the EPDS score is not significantly increased for the mothers with the presence of T allele in the OXTR_rs237885 genotype (Mann–Whitney test, *p* = 0.106) (Table 9).

## 3. Discussion

The aim of this research was to assess the health of mothers’ moods over the first 6 weeks postpartum, using Edinburgh Postnatal Depression Scale, and also, to assess the predictive validity of genetic OXT (rs4813627) and OXTR (rs237885; rs2740210) single nucleotide polymorphisms for the susceptibility to the occurrence of postpartum depression.

Our research is the first known population-based study aiming specifically to provide these outcomes and evaluate the clinical utility of screening mothers in the immediate postpartum period. The current findings of our research are generalizable, since the postpartum sample examined was a representative one of the general population. Furthermore, we applied parallel-processing evaluation for three of the genetic single nucleotide polymorphisms of the oxytocin gene or the oxytocin receptor gene for the occurrence of postpartum depression. This parallel-processing approach aimed to preclude commonly occurring false positive findings which could be due to multiple separate testing.

While postpartum depression is amenable to treatment, preliminary research suggests mothers at risk may be identified early in the postpartum period such that secondary preventive interventions may be implemented [1], for example by using the Edinburgh Postnatal Depression Scale as a strategy for identifying depressive symptoms among postpartum mothers. EPDS is the most widely used self-report tool for screening depression in pregnant and postpartum individuals within primary care settings worldwide [18,19]. The EPDS is recommended to be used as a single-factor scale [20]. Each item has a score on a four-point Likert scale, which can range from 0 (absence of symptoms) to 3 (high severity of symptoms), while the total score can vary between 0 and 30. The selection of the cutoff value is contingent upon the objectives of the assessment; for broad-based screening programs or community surveys, a cutoff value of 9 or 10 is typically deemed most appropriate [21]. Conversely, in clinical environments and research contexts—especially in effectiveness studies where treatment is specifically targeted at individuals most likely to encounter depressive symptoms during the perinatal period—a higher cutoff value of 12 or 13 is recommended [21]. This distinction ensures that the screening and subsequent interventions are tailored effectively to the needs of different populations [22,23].

Oxytocin (OXT), a neuropeptide composed of nine amino acids and produced in the hypothalamus, plays a key role in regulating social behavior and social cognition through its specific receptor, OXTR [6]. There is accumulating evidence for the role of oxytocin in the regulation of human mothering [24]. Increasing evidence suggests that oxytocin plays a role in psychiatric illnesses characterized by social dysfunction and mood disorders [25]. The oxytocin system is influenced by early adverse experiences and has been linked to depression [26]. Plasma oxytocin concentrations during pregnancy were positively linked to a range of maternal bonding behaviors, including positive affect, eye contact during interactions, and cognitive attachment representations towards the newborn in the early postpartum period [27]. In women experiencing postnatal depression, these same behaviors are impaired, along with feelings of being overwhelmed and challenges in developing emotional attachment to their child [28]. Given the significant physiological changes caused by hormonal fluctuations during pregnancy and the abrupt shift following childbirth, it is not yet possible to establish a comprehensive biological model for the development of peripartum depression that accounts for all contributing factors. However, the connection between oxytocin and depression remains unclear [29]. Future studies should also aim to experimentally measure oxytocin concentrations during pregnancy to determine whether oxytocin levels contribute to the development of depressive symptoms in the postpartum period.

Genetic association studies suggest that several oxytocin ligand and receptor genes play a role in the regulation of mothering [6,13]. Another area of research has found that variation in the oxytocin receptor gene (OXTR) is linked to individual differences in social behaviors, early life stress, depression, and anxiety [30]. In fact, gene polymorphisms within the oxytocin system influence the relationship between current stress, past life experiences and social or maternal caregiving behaviors [6,14]. Previous studies examining the OXTR gene in relation to mental health, particularly the interaction effects of the OXTR rs53576 genotype and environment, have primarily focused on adolescents or young adults [31]. One genetic association study identified a link between oxytocin receptor gene polymorphisms and depression [32]. It would be highly valuable to investigate this issue in vulnerable populations, such as postpartum mothers.

Many studies have highlighted disruptions in the oxytocin system in depression, suggesting that exploring its role in maternal depression could be a promising area for research and potential intervention [4]. We examined polymorphic variation in three oxytocin genes: two encoding for the oxytocin peptide (OXT) and one encoding for the oxytocin receptor (OXTR). As far as we know, no studies have assessed the association between the OXTR rs2740210, OXT rs4813627, or OXTR rs237885 polymorphisms and the development of postpartum depression. Therefore, our research is the first known to report on this topic in the literature.

The results obtained showed that polymorphisms in OXT, but not in OXTR, could predict the onset of postpartum depression, and also, could be used as a maternal instrumental care screening tool. The occurrence of postpartum depression was shown only among mothers with the CC genotype of OXT_rs2740210, as we mentioned above, not in those with AA/AC genotypes, supporting the main hypothesis of our study. Among CC homozygotes, as clinical depressive symptomatology manifest in postpartum period, there was an increase in EPDS scores, indicating greater self-reported depression. It is noteworthy that the C allele in the genotype of OXT_rs2740210 is associated with the occurrence of maternal postpartum depression in a subgroup of vulnerable women. One of the possible mechanisms that can explain the present finding could be the interaction between the C allele of OXT_rs2740210 and some risk factors present in this vulnerable period, which may dysregulate the oxytocin system, decreasing, in turn, the resilience to stress, leading to the onset of postpartum depression. The mean value of EPDS score is significantly low for the mothers with presence of A allele in the OXT_rs2740210 genotype. The mean value of EPDS score is not significantly increased for the mothers with the presence of C allele in the OXT_rs2740210 genotype. Thus, the presence of A allele in the genotype of oxytocin gene OXT_rs2740210 could be considered a protective factor for the occurrence of postpartum depression.

Even if there was not observed any significant association between the occurrence of maternal postpartum depression and oxytocin OXT_rs4813627 genotype, our research highlights the fact that the presence of G allele in the genotype of OXT_rs4813627 is associated with the occurrence of maternal postpartum depression in a subgroup of vulnerable woman. These findings are in line with previously observed biological differences, such as lower plasma oxytocin levels in GG homozygotes [13] and the results reported by Costa et al. [32] in an Italian sample, which showed a positive association between the GG genotype of OXTR_rs53576, OXTR_rs2254298, and unipolar depression [5]. Thus, the presence of A allele in the genotype of the oxytocin gene OXT_rs4813627 could be considered a protective factor for the occurrence of postpartum depression. In this sample, mothers with the GG genotype showed high scores on the Edinburgh Postnatal Depression Questionnaire. The mean value of the EPDS score is not significantly low for the mothers with the presence of A allele in the OXT_rs4813627 genotype, and it is not either significantly increased for the mothers with the presence of G allele in the OXT_rs4813627 genotype.

Studying gene-environment interactions in candidate genes may enhance our understanding of individual differences in sensitivity to environmental factors. Much evidence, from studies conducted in the literature, of an association present between different attachment styles and depressive disorders, prompted us to investigate the potential role of polymorphisms within the gene encoding the receptor of oxytocin, the main neurohormone that is implicated in the attachment processes. Similar findings are reported for the OXTR polymorphism rs2254298, which associates with depression and anxiety in adults [32] and, in the face of early adversity, associates with anxiety and depression in adolescent girls [33]. Another OXTR polymorphism, rs53576, has previously been shown to associate with maternal sensitivity and depression [30,34]. We did not find available genotypes for these single nucleotide polymorphisms, but we examined the rs237885 polymorphism, which also lies on the OXTR gene. There was not observed any significant association between maternal postpartum depression and the oxytocin receptor OXTR_rs237885 genotype. Likewise, we found no significant increased interaction effect between mothers with the presence of T or G allele in the genotype of OXTR_rs237885 and the occurrence or not of maternal postpartum depression. The mean value of the EPDS score is not significantly low for the mothers with the presence of G allele in the OXTR_rs237885 genotype, and it is not either significantly increased for the mothers with the presence of T allele in the OXTR_rs237885 genotype.

The findings highlight the importance of considering possible environmental influences in behavior genetic studies and the possibility that contradictory findings on genetic influences on depression could be explained by such environmental factors [5,35]. The examination of both genetic and environmental factors could help and facilitate the identification of women at risk (with genetic predispositions for postpartum depression psychopathology). An emerging field, epigenetics, has been proposed to provide a biological basis for gene–environment interactions [5]. Epigenetics refers to reversible modifications to the DNA sequence at a chromatin level that are not encoded in the DNA and can influence levels of gene expression [36]. Specifically, environmental factors have been suggested to confer a depression risk through epigenetic modifications to the genome [37]. DNA methylation has been found to be responsive to the environment, indicating gene–environment interactions [38]. A recent study also found differences in OXTR DNA methylation between depressed and non-depressed individuals [5]. Decreased OXTR exon 1 methylation was observed in depressed women compared to non-depressed women, and the OXTR rs53576 genotype was found to moderate this association [38]. However, the effect of epigenetics of OXTR on social and emotional behavior in human populations is still a relatively new field of inquiry pending firmly conclusive evidence [39]. The three-way genetic interaction in the present research also has a contribution to the growing evidence of gene–depression interactions, which could highlight the importance of taking genetics studies into consideration when facedwith a group of vulnerable women. In addition, these results may differ across populations and cultures, suggesting that the interaction effects between postpartum depression and OXT/OXTR SNP genotypes may also depend on the population; therefore, there may be other different target genes to be considered. Thus, the generalization of the results across different populations from across the world should be investigated and considered by replicating the present study across other populations and taking into consideration a broader spectrum of target genes of interest.

There are some limitations of this research that should be acknowledged. Firstly, we used self-report measures, and the study was conducted in a clinical sample, at the consultation scheduled 6 weeks after childbirth. Longitudinal studies with replication of this research with other clinical population samples from all over the world, would provide a clearer image of the association between postpartum depression and single nucleotide polymorphisms in the oxytocin/oxytocin receptor gene. Secondly, while there were found associations between postpartum depression and mothers with the CC genotype of OXT_rs2740210 and also the presence of C allele in the genotype, the mechanisms that mediate this relationship have not yet been elucidated. Future studies could investigate some biological markers like plasma oxytocin levels, to determine if there are any significant mediators implicated. Another limitation that we have to mention concerns the size of the population sample (n = 100), which is relatively small, but nevertheless sufficient to detect significant effects. The sample size could limit statistical power and may also increase the risk of false positive findings. However, future research should verify the replicability of our results by considering larger postpartum women samples from different populations.

Considering the moderate heritability of depression and evidence suggesting the interaction between genes and the environment in its development [40], further research is necessary to investigate the role of candidate genes in the intergenerational transmission of depressive disorder. Early detection and closer monitoring of women with specific OXT genetic variants, along with other risk factors, could help mitigate the long-term effects of peripartum depression.

## 4. Materials and Methods

### 4.1. Participants

A total of 100 postpartum mothers were recruited in the study, ranging from 18 to 46 years old (mean 29.17). All participants were investigated individually at the consultation scheduled 6 weeks after childbirth. The distribution of socio-demographic characteristics, obstetric indicators, and other descriptive data among study participants are presented in Table 10.

### 4.2. Ethics Declarations

The authorized Local Commission on the Ethics for Scientific Research from Timis County Emergency Clinical Hospital “Pius Brînzeu”, Timisoara, Romania, as an advisory body, operates under article 167 provisions of Law no. 95/2006, art. 28, chapter VIII of order 904/2006 and with EU GCP Directives 2005/28/EC, International Conference of Harmonisation of Technical Requirements for Registration of Pharmaceuticals for Human Use (ICH), and with the Declaration of Helsinki—Recommendations Guiding Medical Doctors in Biomedical Research Involving Human Subjects. The current research was conducted in accordance with the guidelines of the Declaration of Helsinki, in compliance of the European Union General Data Protection Regulation (GDPR), and also, was approved by the authorized Local Commission of Ethics for Scientific Research from the Timis County Emergency Clinical Hospital “Pius Brinzeu”, Timisoara, Romania, by No. 423/04.12.2023.

### 4.3. Procedure

A case–control design study was used to enroll 100 postpartum participants. For the purpose of our research, postpartum mothers were meticulously screened and enrolled in the study based on a well-established set of inclusion/exclusion criteria to ensure the specificity and sensitivity of our participant pool. The study took place in the Obstetrics and Gynecology Clinical Section I of the “Pius Brînzeu” County Emergency Hospital from Timisoara, Romania. Prior written informed consent was obtained from each participant incorporated into the investigation, as it includes sensitive data and also a vulnerable group of women.

- Participants were included in the study if they met the following inclusion criteria:
- Postpartum mothers within the age range of 18–50 years.
- Postpartum mothers at the consultation scheduled 6 weeks after childbirth.
- No history of high-risk obstetrical pregnancy in the case of the last pregnancy (gestational diabetes, pregnancy induced hypertension, preeclampsia, intrahepatic cholestasis of pregnancy, fetal anomalies, chromosomal aberrations, intrauterine growth restriction).
- No antepartum/postpartum dead fetus.
- Postpartum mothers who expressed an interest in this subject and who also have provided informed consent to be enrolled in the study.

Postpartum mothers were excluded if they met any of the following exclusion criteria:

- Mothers with current or past use of any sort of psychotropic medication.
- Mothers with high-risk obstetrical pregnancy in the case of the last one (gestational diabetes, pregnancy induced hypertension, preeclampsia, intrahepatic cholestasis of pregnancy, fetal anomalies, chromosomal aberrations, intrauterine growth restriction).
- Mothers with a history of psychiatric disorders or any mental health issues.
- Patients with twin, triplet, or higher-order multifetal gestations.

Postpartum depression screening involves the use of self-report questionnaires, which are used to assess symptoms of postpartum depression and identify mothers with a score above a preidentified cut-off value to determine whether postpartum depression is present or not. The Edinburgh Postnatal Depression Scale (EPDS) questionnaire was completed at 6 weeks after childbirth by the mothers who met the inclusion criteria. With the aim of highlighting the risk factors and particularities of postpartum depression, the following parameters of included participants were also taken into account: age at delivery, residence, education, marital status, socio-economic conditions, workplace hazard, health status, gravidity, parity, number of miscarriages, number of abortions performed upon request, personal pathological history (psychiatric disorders, postpartum depressive disorder), history of psychotherapy, current known depressive disorder, method of achieving pregnancy, type of birth, preterm birth, the presence of insomnia, anxiety, family history of psychiatric disorders.

### 4.4. Edinburgh Postnatal Depression Scale Questionnaire

The Edinburgh Postnatal Depression Scale (EPDS) has established psychometric properties and is the most widely used scale to screen for perinatal depression [41] and has been used globally for over three decades [42,43]. The EPDS is a 10-question unidimensional self-report scale that uses a 4-point Likert scale with response categories ranging from 0 to 3 according to increasing severity of symptom [44]. The scores can range from 0 to 30 [44]. Previous research has revealed that the sensitivity of the EPDS for true positives for postpartum depressed women is 86%, and specificity for true negatives for non-depressed women is 78% [45]. The cutoff point used to identify women as high risk for postpartum depression varies, with most studies using a cutoff score of ≥10 or ≥12 [46]. A cutoff score ≥10 detects a major depressive disorder with a sensitivity of >90% and specificity >80%, and a cutoff score ≥13 detects a major depressive disorder with sensitivity of >85% and specificity of >80% [46,47]. In our study, we used a cutoff score of ≥13 to detect postpartum depression. EPDS is widely used in international clinical and research work, has been translated into over sixty languages, validated in most regions of the world, and is recommended as a useful adjunct to the assessment of perinatal women [42]. With a threshold of 9, the Edinburgh Postnatal Depression Scale has a satisfactory power to detect women complaining of a depressive episode between 4 and 8 weeks postpartum and is therefore considered a good tool for screening of PPD in community settings [48].

### 4.5. DNA Genotyping and Allelic Discrimination

DNA was extracted from peripheral blood samples of the participants (N = 100) and evaluated for the OXT (rs4813627) and OXTR (rs237885; rs2740210) single nucleotide polymorphisms. Genomic DNA was isolated from blood lymphocytes using a PureLink Genomic DNA Mini Kit (Invitrogen, Waltham, MA, USA) according to manufacturer protocol. Genotyping was conducted on the QuantStudio 7 Flex Real-Time PCR System (Applied Biosystems, Waltham, MA, USA). For this study, one variant (rs237885) of the OXTR gene and two variants (rs2740210 and rs4813627) of the OXT gene were selected for evaluation. TaqMan Genotyping Assays (Applied Biosystems Assay ID: C_3290319_1_, C_16061225_10, C_2712196_10) and TaqMan Genotyping Master Mix (Applied Biosystems, Waltham, MA, USA) were utilized according to the manufacturer’s protocol. We randomly re-genotyped 10% of the samples for quality control purposes.

### 4.6. Statistical Analysis

The Statistical JASP v0.18.3 (University of Amsterdam) software was used to analyze the data recorded in Microsoft Excel. Qualitative variables were described as percentages, while quantitative variables as mean ± standard deviation (SD). The Mann–Whitney test was used to observe differences between two study groups, the Kruskal-Wallis test was applied for comparison between more than two study groups, and the Chi-Square test was used to analyze the categorical data. A *p* value < 0.05 was considered statistically significant. We assessed the presence of major depressive symptomatology (EPDS ≥ 13) and calculated the frequencies of every possible risk factor in the total sample.

## 5. Conclusions

Postpartum depression is a leading cause of disability affecting women and could lead to adverse outcomes for the developing child. However, most mothers with peripartum depression do not receive adequate and enough care, making it a critical clinical priority to improve support for postpartum women with depression.

The present study has proposed an investigation into gene–environment interaction effects for the occurrence of postpartum depression and OXT SNP genotypes of rs2740210; rs4813627 and OXTR SNP genotypes of s237885. In sum, our research findings support hypotheses about the involvement of oxytocinergic gene variants in vulnerability for postpartum depression. The results highlighted a significant interaction between the OXT_rs2740210 genotype and maternal postpartum depression in mothers with the CC genotype. This reveals that an interaction of vulnerable genotypes (CC genotype of OXT_rs2740210, C allele in genotype of OXT_rs2740210, G allele in genotype of OXT_rs4813627) with an environmental burden or other risk factors, would predispose the mothers to develop postpartum depression. Hence, special attention needs to be paid to mothers exposed to risk factors for maternal postpartum depression in order to perform an effective screening and support the provision of appropriate care.

While the direct clinical implications of behavior-genetics studies can be challenging and often difficult to determine, we found a significant association between oxytocin pathway gene variants and the diagnosis of postpartum depression disorder. Our findings prove the implication of the oxytocinergic system in the pathophysiological mechanisms that underlie postpartum depression among women possessing the CC genotype in the genetic variation in the oxytocin peptide gene OXT_rs2740210. Future research should focus on exploring and further understanding the relationship between the oxytocin OXT_rs2740210 genetic variation and its role in the onset of peripartum depression.

## Figures and Tables

**Table 1:** The genotype distributions for OXT_rs2740210 depending on the occurrence of postpartum depression and other variables of study participants (N = 98).

Variable	OXT_rs2740210 Genotype			*p* ^Test^
	AA (13)	AC (41)	CC (44)	
**Presence of PPD**				
No	13 (15.500%)	41 (48.800%)	30 (35.700%)	<0.001 ^(C)^ *
Yes	0 (0.000%)	0 (0.000%)	14 (87.500%)	
**EPDS score**	8.462 ± 4.054 11 (5–11)	9.317 ± 3.365 11 (8–11)	10.455 ± 4.547 11 (6–13)	0.213 ^(K-W)^
**Marital status**				
Cohabiting	2 (18.182%)	6 (54.545%)	3 (27.273%)	0.312 ^(C)^
Divorced	0 (0.000%)	2 (66.667%)	1 (33.333%)	
Married	9 (11.842%)	28 (36.842%)	39 (51.316%)	
Single	2 (25.000%)	5 (62.500%)	1 (12.500%)	
**Residence**				
Rural	7 (11.864%)	24 (40.678%)	28 (47.458%)	0.785 ^(C)^
Urban	6 (15.385%)	17 (43.590%)	16 (41.026%)	
**Education**				
High school	3 (10.000%)	11 (36.667%)	16 (53.333%)	0.541 ^(C)^
Higher education	5 (10.417%)	21 (43.750%)	22 (45.833%)	
Middle school	3 (25.000%)	6 (50.000%)	3 (25.000%)	
Primary education	1 (50.000%)	0 (0.000%)	1 (50.000%)	
Vocational school	1 (16.667%)	3 (50.000%)	2 (33.333%)	
**Socio-economic conditions**				
Good standard of living	8 (14.286%)	24 (42.857%)	24 (42.857%)	0.112 ^(C)^
Poor living conditions	0 (0.000%)	0 (0.000%)	1 (100.000%)	
Satisfactory conditions	4 (19.048%)	12 (57.143%)	5 (23.810%)	
Very good standard of living	1 (5.000%)	5 (25.000%)	14 (70.000%)	
**Health status**				
Good	11 (11.957%)	39 (42.391%)	42 (45.652%)	0.326 ^(C)^
Satisfactory	2 (33.333%)	2 (33.333%)	2 (33.333%)	
**Type of birth**				
Caesarean section	7 (11.475%)	24 (39.344%)	30 (49.180%)	0.525 ^(C)^
Vaginal delivery	6 (16.216%)	17 (45.946%)	14 (37.838%)	
**Preterm birth**				
No	12 (14.634%)	32 (39.024%)	38 (46.341%)	0.388 ^(C)^
Yes	1 (6.250%)	9 (56.250%)	6 (37.500%)	
**History of depressive disorder**				
No	11 (11.702%)	40 (42.553%)	43 (45.745%)	0.087 ^(C)^
Yes	2 (50.000%)	1 (25.000%)	1 (25.000%)	
**History of postpartum depressive disorder**				
No	12 (12.903%)	40 (43.011%)	41 (44.086%)	0.592 ^(C)^
Yes	1 (20.000%)	1 (20.000%)	3 (60.000%)	
**Insomnia**				
No	9 (12.000%)	33 (44.000%)	33 (44.000%)	0.670 ^(C)^
Yes	4 (17.391%)	8 (34.783%)	11 (47.826%)	
**Anxiety**				
No	11 (14.474%)	35 (46.053%)	30 (39.474%)	0.133 ^(C)^
Yes	2 (9.091%)	6 (27.273%)	14 (63.636%)	
**Family history of psychiatric disorders**				
No	13 (13.542%)	40 (41.667%)	43 (44.792%)	0.854 ^(C)^
Yes	0 (0.000%)	1 (50.000%)	1 (50.000%)	
**History of psychotherapy**				
No	12 (12.500%)	41 (42.708%)	43 (44.792%)	0.230 ^(C)^
Yes	1 (50.000%)	0 (0.000%)	1 (50.000%)	

*—significant association; (C)—Chi2 test; (K-W)—Kruskal-Wallis test.

**Table 2:** Presence of A and C alleles in the OXT_rs2740210 genotype depending on the occurrence of postpartum depression.

PPD		Presence of A Allele in the OXT_rs2740210 Genotype			Total	Presence of C Allele in the OXT_rs2740210 Genotype			Total
		NA = No Results	No	Yes		NA = No Results	No	Yes	
**With PPD**	Count	2	14	0	16	2	0	14	16
	% within PPD	12.5%	87.5%	0.0%	100.0%	12.5%	0.0%	87.5%	100.0%
**Without PPD**	Count	0	30	54	84	0	13	71	84
	% within PPD	0.0%	35.7%	64.3%	100.0%	0.0%	15.5%	84.5%	100.0%
**Total**	Count	2	44	54	100	2	13	85	100
	% within PPD	2.0%	44.0%	54.0%	100.0%	2.0%	13.0%	85.0%	100.0%

**Table 3:** Presence of A and C alleles in the OXT_rs2740210 genotype depending on EPDS score.

	Group	N	Mean EPDS SCORE	SD	SE	Coefficient of Variation
**Presence of A allele in OXT_rs2740210 genotype**	**No**	44	10.455	4.547	0.685	0.435
	**Yes**	54	9.111	3.522	0.479	0.387
**Presence of C allele in OXT_rs2740210 genotype**	**No**	13	8.462	4.054	1.124	0.479
	**Yes**	85	9.906	4.037	0.438	0.408

**Table 4:** The genotype distributions for the OXT_rs4813627 depending on the occurrence of postpartum depression and other variables of the study participants (N = 99).

Variable	OXT_rs4813627 Genotype			*p* ^Test^
	AA (24)	AG (52)	GG (23)	
**Presence of PPD**				
No	22 (26.200%)	45 (53.600%)	17 (20.200%)	0.050 ^(C)^
Yes	2 (12.500%)	7 (43.800%)	6 (37.500%)	
**EPDS Score**	9.208 ± 4.139 11 (6–11)	9.788 ± 3.455 11 (8–11)	10.217 ± 5.161 11 (6.5–12.5)	0.694 ^(K-W)^
**Marital status**				
Cohabiting	1 (9.091%)	7 (63.636%)	3 (27.273%)	0.520 ^(C)^
Divorced	2 (66.667%)	1 (33.333%)	0 (0.000%)	
Married	19 (24.675%)	39 (50.649%)	19 (24.675%)	
Single	2 (25.000%)	5 (62.500%)	1 (12.500%)	
**Residence**				
Rural	15 (25.424%)	29 (49.153%)	15 (25.424%)	0.704 ^(C)^
Urban	9 (22.500%)	23 (57.500%)	8 (20.000%)	
**Education**				
High school	8 (26.667%)	13 (43.333%)	9 (30.000%)	0.914 ^(C)^
Higher education	11 (22.449%)	28 (57.143%)	10 (20.408%)	
Middle school	3 (25.000%)	7 (58.333%)	2 (16.667%)	
Primary education	0 (0.000%)	1 (50.000%)	1 (50.000%)	
Vocational school	2 (33.333%)	3 (50.000%)	1 (16.667%)	
**Socio-economic conditions**				
Good standard of living	15 (26.316%)	30 (52.632%)	12 (21.053%)	0.559 ^(C)^
Poor living conditions	0 (0.000%)	0 (0.000%)	1 (100.000%)	
Satisfactory conditions	4 (19.048%)	13 (61.905%)	4 (19.048%)	
Very good standard of living	5 (25.000%)	9 (45.000%)	6 (30.000%)	
**Health status**				
Good	22 (23.656%)	48 (51.613%)	23 (24.731%)	0.378 ^(C)^
Satisfactory	2 (33.333%)	4 (66.667%)	0 (0.000%)	
**Type of birth**				
Caesarean section	12 (19.355%)	35 (56.452%)	15 (24.194%)	0.335 ^(C)^
Vaginal delivery	12 (32.432%)	17 (45.946%)	8 (21.622%)	
**Preterm birth**				
No	22 (26.506%)	42 (50.602%)	19 (22.892%)	0.479 ^(C)^
Yes	2 (12.500%)	10 (62.500%)	4 (25.000%)	
**History of depressive disorder**				
No	22 (23.158%)	51 (53.684%)	22 (23.158%)	0.417 ^(C)^
Yes	2 (50.000%)	1 (25.000%)	1 (25.000%)	
**History of postpartum depressive disorder**				
No	22 (23.404%)	52 (55.319%)	20 (21.277%)	0.041 ^(C)^ *
Yes	2 (40.000%)	0 (0.000%)	3 (60.000%)	
**Insomnia**				
No	18 (24.000%)	38 (50.667%)	19 (25.333%)	0.671 ^(C)^
Yes	6 (25.000%)	14 (58.333%)	4 (16.667%)	
**Anxiety**				
No	17 (22.368%)	42 (55.263%)	17 (22.368%)	0.593 ^(C)^
Yes	7 (30.435%)	10 (43.478%)	6 (26.087%)	
**Family history of psychiatric disorders**				
No	24 (24.742%)	50 (51.546%)	23 (23.711%)	0.398 ^(C)^
Yes	0 (0.000%)	2 (100.000%)	0 (0.000%)	
**History of psychotherapy**				
No	23 (23.711%)	52 (53.608%)	22 (22.680%)	0.323 ^(C)^
Yes	1 (50.000%)	0 (0.000%)	1 (50.000%)	

*—significant association; (C)—Chi2 test; (K-W)—Kruskal-Wallis test.

**Table 5:** Presence of A and G alleles in the OXT_rs4813627 genotype depending on the occurrence of postpartum depression.

PPD		Presence of A Allele in OXT_rs4813627 Genotype			Total	Presence of G Allele in OXT_rs4813627 Genotype			Total
		NA = No Results	No	Yes		NA = No Results	No	Yes	
**With PPD**	Count	1	6	9	16	1	2	13	16
	% within PPD	6.3%	37.5%	56.3%	100.0%	6.3%	12.5%	81.3%	100.0%
**Without PPD**	Count	0	17	67	84	0	22	62	84
	% within PPD	0.0%	20.2%	79.8%	100.0%	0.0%	26.2%	73.8%	100.0%
**Total**	Count	1	23	76	100	1	24	75	100
	% within PPD	1.0%	23.0%	76.0%	100.0%	1.0%	24.0%	75.0%	100.0%

**Table 6:** Presence of A and G alleles in the OXT_rs4813627 genotype depending on EPDS score.

	Group	N	Mean EPDS SCORE	SD	SE	Coefficient of Variation
**Presence of A allele in OXT_rs4813627 genotype**	**No**	23	10.217	5.161	1.076	0.505
	**Yes**	76	9.605	3.667	0.421	0.382
**Presence of G allele in OXT_rs4813627 genotype**	**No**	24	9.208	4.139	0.845	0.449
	**Yes**	75	9.92	4.023	0.465	0.406

**Table 7:** The genotype distributions for OXTR_rs237885 depending on the occurrence of postpartum depression and other variables of study participants (N = 97).

Variable	OXTR_rs237885 Genotype			*p* ^Test^
	GG (37)	GT (45)	TT (15)	
**Presence of PPD**				
No	32 (38.100%)	37 (44.000%)	13 (15.500%)	0.795 ^(C)^
Yes	5 (31.300%)	8 (50.000%)	2 (12.500%)	
**EPDS score**	9.081 ± 4.468 11 (5–11)	10.111 ± 4.013 11 (8–12)	10.467 ± 3.021 11 (10.5–12)	0.406 ^(K-W)^
**Marital status**				
Cohabiting	5 (45.455%)	4 (36.364%)	2 (18.182%)	0.820 ^(C)^
Divorced	1 (33.333%)	1 (33.333%)	1 (33.333%)	
Married	28 (37.333%)	35 (46.667%)	12 (16.000%)	
Single	3 (37.500%)	5 (62.500%)	0 (0.000%)	
**Residence**				
Rural	26 (44.828%)	26 (44.828%)	6 (10.345%)	0.122 ^(C)^
Urban	11 (28.205%)	19 (48.718%)	9 (23.077%)	
**Education**				
High school	11 (39.286%)	11 (39.286%)	6 (21.429%)	0.765 ^(C)^
Higher education	19 (38.776%)	22 (44.898%)	8 (16.327%)	
Middle school	5 (41.667%)	7 (58.333%)	0 (0.000%)	
Primary education	1 (50.000%)	1 (50.000%)	0 (0.000%)	
Vocational school	1 (16.667%)	4 (66.667%)	1 (16.667%)	
**Socio-economic conditions**				
Good standard of living	24 (42.105%)	26 (45.614%)	7 (12.281%)	0.665 ^(C)^
Poor living conditions	0 (0.000%)	1 (100.000%)	0 (0.000%)	
Satisfactory conditions	8 (38.095%)	10 (47.619%)	3 (14.286%)	
Very good standard of living	5 (27.778%)	8 (44.444%)	5 (27.778%)	
**Health status**				
Good	37 (40.659%)	41 (45.055%)	13 (14.286%)	0.115 ^(C)^
Satisfactory	0 (0.000%)	4 (66.667%)	2 (33.333%)	
**Type of birth**				
Caesarean section	23 (37.705%)	27 (44.262%)	11 (18.033%)	0.647 ^(C)^
Vaginal delivery	14 (38.889%)	18 (50.000%)	4 (11.111%)	
**Preterm birth**				
No	31 (38.272%)	36 (44.444%)	14 (17.284%)	0.483 ^(C)^
Yes	6 (37.500%)	9 (56.250%)	1 (6.250%)	
**History of depressive disorder**				
No	36 (38.710%)	43 (46.237%)	14 (15.054%)	0.800 ^(C)^
Yes	1 (25.000%)	2 (50.000%)	1 (25.000%)	
**History of postpartum depressive disorder**				
No	36 (39.130%)	43 (46.739%)	13 (14.130%)	0.279 ^(C)^
Yes	1 (20.000%)	2 (40.000%)	2 (40.000%)	
**Insomnia**				
No	29 (39.726%)	34 (46.575%)	10 (13.699%)	0.674 ^(C)^
Yes	8 (33.333%)	11 (45.833%)	5 (20.833%)	
**Anxiety**				
No	30 (40.541%)	36 (48.649%)	8 (10.811%)	0.075 ^(C)^
Yes	7 (30.435%)	9 (39.130%)	7 (30.435%)	
**Family history of psychiatric disorders**				
No	36 (37.895%)	44 (46.316%)	15 (15.789%)	0.820 ^(C)^
Yes	1 (50.000%)	1 (50.000%)	0 (0.000%)	
**History of psychotherapy**				
No	36 (37.895%)	44 (46.316%)	15 (15.789%)	0.820 ^(C)^
Yes	1 (50.000%)	1 (50.000%)	0 (0.000%)	

^(C)^—Chi2 test; ^(K-W)^—Kruskal-Wallis test.

**Table 8:** Presence of G and T alleles in the OXTR_rs237885 genotype depending on the occurrence of postpartum depression.

PPD		Presence of G Allele in OXTR_rs237885 Genotype			Total	Presence of T Allele in OXTR_rs237885 Genotype			Total
		NA = No results	No	Yes		NA = No Results	No	Yes	
**With PPD**	Count	1	2	13	16	1	5	10	16
	% within PPD	6.3%	12.5%	81.3%	100.0%	6.3%	31.3%	62.5%	100.0%
**Without PPD**	Count	2	13	69	84	2	32	50	84
	% within PPD	2.4%	15.5%	82.1%	100.0%	2.4%	38.1%	59.5%	100.0%
**Total**	Count	3	15	82	100	3	37	60	100
	% within PPD	3.0%	15.0%	82.0%	100.0%	3.0%	37.0%	60.0%	100.0%

**Table 9:** Presence of G and T alleles in the OXTR_rs237885 genotype depending on EPDS score.

	Group	N	Mean EPDS SCORE	SD	SE	Coefficient of Variation
**Presence of G allele in OXTR_rs237885 genotype**	**No**	15	10.467	3.021	0.78	0.289
	**Yes**	82	9.646	4.229	0.467	0.438
**Presence of T allele in OXTR_rs237885 genotype**	**No**	37	9.081	4.468	0.735	0.492
	**Yes**	60	10.2	3.768	0.486	0.369

**Table 10:** Socio-demographic, obstetric indicators, and other descriptive data among study participants (N = 100).

Age	
Range	18–46
Mean ± Standard Deviation	29.17 ± 6.08
***Residence***	
Rural	60% (60)
Urban	40% (40)
***Education***	
High School	31% (31)
Higher education	49% (49)
Middle school	12% (12)
Primary education	2% (2)
Vocational school	6% (6)
***Socio-economic conditions***	
Poor living conditions	1% (1)
Satisfactory conditions	21% (21)
Good standard of living	57% (57)
Very good standard of living	21% (21)
***Workplace hazard***	
High	5% (5)
Medium	15% (15)
Low	80% (80)
***Health status***	
Good	94% (94)
Satisfactory	6% (6)
***Gravidity***	
1	43% (43)
2	26% (26)
3	15% (15)
4	7% (7)
5	6% (6)
6	1% (1)
7	1% (1)
9	1% (1)
***Parity***	
1	55% (55)
2	26% (26)
3	12% (12)
4	2% (2)
5	2% (2)
6	3% (3)
***Miscarriages***	
0	81% (81)
1	14% (14)
2	3% (3)
3	2% (2)
***Abortions performed upon request***	
0	87% (87)
1	11% (11)
2	2% (2)
***Method of achieving pregnancy***	
In vitro fertilization	1% (1)
Naturally	98% (98)
With previous treatment	1% (1)
***Type of birth***	
Caesarean section	63% (63)
Vaginal delivery	37% (37)
***Preterm birth***	
No	83% (83)
Yes	17% (17)
***Psychiatric disorders***	
No	100% (100)
***Current depressive disorder***	
No	100% (100)
***History of depressive disorder***	
No	96% (96)
Yes	4% (4)
***History of postpartum depressive disorder***	
No	95% (95)
Yes	5% (5)
***Insomnia***	
No	75% (75)
Yes	25% (25)
***Anxiety***	
No	76% (76)
Yes	24% (24)
***Family history of psychiatric disorders***	
No	98% (98)
Yes	2% (2)
***History of psychotherapy***	
No	98% (98)
Yes	2% (2)

## Data Availability

The original contributions presented in the study are included in the article; further inquiries can be directed to the corresponding author.

## Abbreviations

The following abbreviations are used in this manuscript:

PPD	Postpartum depression
EPDS	Edinburgh Postnatal Depression Scale
OXT	Oxytocin
OXTR	Oxytocin receptor
SNPs	Single nucleotide polymorphisms
DNA	Deoxyribonucleic acid
